# Carbopol emulgel loaded with ebastine for urticaria: development, characterization, *in vitro* and *in vivo* evaluation

**DOI:** 10.1080/10717544.2021.2015483

**Published:** 2021-12-28

**Authors:** Barkat Ali Khan, Arshad Ali, Khaled M. Hosny, Abdulrahman A. Halwani, Alshaimaa M. Almehmady, Muhammad Iqbal, Waleed S. Alharbi, Walaa A. Abualsunun, Rana B. Bakhaidar, Samar S. A. Murshid, Muhammad Khalid Khan

**Affiliations:** a Drug Delivery and Cosmetic Lab (DDCL), Faculty of Pharmacy, Gomal University, Dera Ismail Khan, Pakistan; b Department of Pharmaceutics, Faculty of Pharmacy, King Abdulaziz University, Jeddah, Saudi Arabia; c Department of Natural Products and Alternative Medicine, Faculty of Pharmacy, King Abdulaziz University, Jeddah, Saudi Arabia

**Keywords:** Urticaria, ebastine, emulgel, anti-allergy, Carbopol

## Abstract

Urticaria affects all age groups of a population. It is triggered by allergens in foods, insect bites, medications, and environmental conditions. Urticaria is characterized by itching, a burning sensation, wheals and flares, erythema, and localized edema. The aim of this study was to develop a polymeric dosage form of ebastine using Carbopol 940 and mixture of span and tween. The emulsion was prepared, the gelling agent was added and the desired emulgel loaded with active drug was formulated. The formulations were subjected to physical stability, pH, viscosity, spreadability, drug content analysis, thermal analysis, *in vitro* drug release, and *in vivo* anti-allergic activity in animal model. The formulated emulgel exhibited good physical stability. The pH of the formulation was in the range of 5.2 ± 0.17 to 5.5 ± 0.20 which is suitable for topical application. Insignificant changes (*p* > .05) were observed in viscosity and spreadability of stored emulgels. The drug content was in the official limit of Pharmacopeia (i.e. 100 ± 10%). DSC measurements predicted that there is no interaction between the active moiety and excipients in emulgel formulation. The optimized formulation (ES3) released 74.25 ± 1.8% of ebastine after 12 h. The ebastine emulgel showed significant (*p* < .05; ANOVA) *in vivo* anti-allergic activity as compared to commercial product Benadryl^®^ in histamine-induced allergy in rabbits. This study concluded that a topical drug delivery of ebastine-loaded emulgel could be well tolerated and safe for the treatment of urticaria/hives.

## Introduction

Currently, topical drug delivery systems are getting more attention among researchers due to their promising therapeutic outcomes for the treatment of local and systemic diseases (Yu et al., 2021). The topical delivery of drugs has many advantages over other dosage forms because it minimizes systemic side effects, prevents metabolism of a drug by the liver, avoids gastrointestinal disorders associated with oral forms, and avoids the inconvenience of parenteral forms (Yamaguchi et al., 1994; Hampel et al., 2002; Yu et al., 2021).

Urticaria is common all over the world and approximately 12–22% of the population may have urticaria at least once in their life span (Liu et al., 2006). The distribution of urticaria in men and women varies among different studies, but it tends to be more prevalent in women than men, i.e. 31–53% (Mahajan & Basarkar, 2019). Urticaria is a cutaneous syndrome characterized by dermal edema (wheals) and erythema, which produces itching or burning sensations. The lesions range from 1 mm to 10 cm and are profoundly itchy, stimulated by skin contact, come and go and may be associated with angioedema (localized swelling of the eyes, lips, mouth, tongue, genitalia, extremities, and bowel wall) (Kamisetti & Vankadari, 2017).

Histamine is one of the prominent mediators of most forms of urticaria; therefore, antihistamines (especially H_1_ antihistamines) are usually used for the treatment of patients with chronic urticaria (Harmalkar et al., 2019; Mehetre et al., 2019). Clinically, urticaria is found in all age groups. The wheals and flares develop within hours (or even sooner). The hives occur in episodes depending on the allergen and may last for a day or for weeks or months (Bhasha, 2013). Three kinds of triggers alter the allergic reaction or (urticaria) hives: (1) foods, including food additives, berries, nuts, eggs, sea foods, and dairy products, (2) medications (e.g. penicillin, sulfas, salicylate, vaccines, and anesthetics), and (3) environmental conditions (e.g. hot and cool weather, exercise, bee or wasp stings) (Paul et al., 1987).

**Figure 1.**
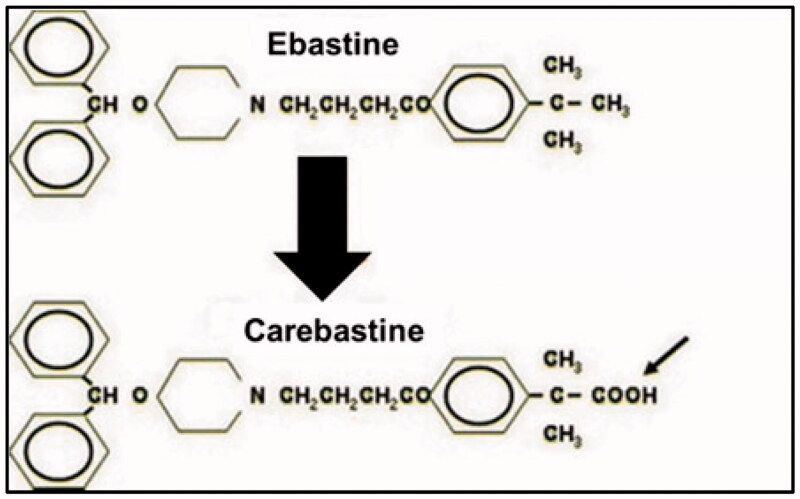
Structure of ebastine and its active metabolite.

Ebastine is a non-sedating, second-generation and long-acting histamine H_1_ receptor antagonist with an oxypiperidine-based structure that is indicated for the treatment of allergic rhinitis and chronic idiopathic urticaria, and in some countries for relief from mosquito bites or atopic dermatitis (Majumder et al., 2015). It can be administered orally in the dose of 10–20 mg daily having long duration of action (*T*_1/2_ about 15 h). It belongs to BCS class II drugs presenting low aqueous solubility and poor oral bioavailability. After oral administration, ebastine is rapidly absorbed but undergoes first pass metabolism that results in producing its active metabolites carebastine and consequently plasma concentrations of ebastine are extremely low (Figure 1) (Ormerod, 1994). Ebastine is a highly protein bound drug, and its maximum plasma concentration (*C*_max_) is achieved in 2–4 hours after administration. Its plasma level increases up to 1.5–2 times when administered with food. By developing an optimized topical formulation, the extensive metabolism of ebastine can be avoided and maximum therapeutic effects can be achieved in diseases such as urticaria (Tanwar & Jain, 2012).

A topical drug delivery system is used frequently for many skin disorders, including local skin infections, wounds, and allergies. It is especially useful when other routes are less effective or can be associated with severe systemic side effects (Tanwar & Jain, 2012). Several dosage forms are available for topical applications, such as creams, ointments, gels, lotions, and emulgels. However, an emulgel has several advantages. For instance, it is hydrophilic as well as hydrophobic; it can be delivered via an emulgel system; and it is more stable than other topical formulations such as creams, which can break down, and ointments, which can become rancid (Hosny et al., 2019). Additionally, it avoids a first-pass effect, easy to apply, avoids the inconveniences associated with intravenous therapy, avoids fluctuations in drug levels, and can deliver drugs selectively to a targeted area. As it is a noninvasive technique, it eliminates the need for nursing and hospitalization and improves patient compliance (Dannert et al., 2019).

Different anti-allergic medications are available in the market place for the treatment of urticaria. An H1 antagonist or antihistamine is the drug of choice for urticaria (Paul et al., 1987). Most antihistamine preparations are available in an oral form.

The focus of this study was to formulate an ebastine-loaded emulgel as a topical delivery system for local urticaria, in order to avoid sedation due to systemic antihistamine therapy and to minimize other side effects associated with oral medications.

## Materials and methods

### Materials

Liquid paraffin was purchased from Merck (Darmstadt, Germany). Carbopol 940 was purchased from Sigma-Aldrich (Darmstadt, Germany). Tween 80 was purchased from Sigma-Aldrich (Darmstadt, Germany). Span 80 was purchased from Sigma-Aldrich (Darmstadt, Germany). Propylene glycol was purchased from Sigma-Aldrich (Darmstadt, Germany). Methylparaben was purchased from Merck (Darmstadt, Germany). Triethanolamine was purchased from Biolife (Varanasi, India). Ethanol was purchased from Merck KGaA (Darmstadt, Germany). Methyl alcohol was purchased from Samchun (Pyeongtaek, Republic of Korea). Distilled water was purchased from Faculty of Pharmacy, Gomal University (Dera Ismail Khan, Pakistan). Ebastine was kindly donated by Medicraft Pharmaceuticals (Peshawar, Pakistan).

### Methods

#### Preparation of ebastine emulgel

The emulgels were prepared according to the study of Burki et al. (2020) with slight modifications. Different formulations of ebastine emulgel were prepared in a two-step process. In the first step, a gel was prepared by dispersing Carbopol 940 in distilled water with constant stirring (at 3000 rpm), and kept overnight with moderate stirring to form a swelled gel network. The pH was adjusted up to 5.5–6.5 by adding a few drops of triethanolamine. In the second step, an emulsion was prepared by mixing the oil phase and aqueous phase. The oil phase consisted of liquid paraffin and Span 80, and the aqueous phase was made by mixing Tween 80 into distilled water. The oil phase and aqueous phase were heated separately at 60–70 °C. The drug was dissolved in methanol, and methylparaben (preservative) was dissolved in propylene glycol. Then, both solutions were added to the aqueous phase and mixed with the help of a magnetic stirrer. Subsequently, the oil phase was added into the aqueous phase solution in a drop wise manner with constant stirring in an electric mixer at 3000 rpm for 10 min. It was then cooled down to room temperature. Finally, both the gel and the emulsion were mixed in 1:1 ratio with continuous stirring at 2000 rpm for 15 minutes. Several formulations of emulgels were prepared with variable amounts of ingredients as shown in Tables 1 and 2.

**Table 1.** Composition of various emulsion formulations (w/w).

Ingredients	ES1	ES2	ES3	ES4	ES5	ES6	ES7	ES8
Ebastine (active drug)	–	1	1	1	1	1	1	1
Liquid paraffin	7	6.5	7	7	5	6	7	6.5
Tween 80	0.6	0.5	0.6	0.6	0.6	0.4	0.6	0.7
Span 80	1	0.8	1	1	0.6	0.8	1	1
Propylene glycol	7	7	7	7	7	7	7	7
Methyl paraben	0.02	0.02	0.02	0.02	0.02	0.02	0.02	0.02
Distilled water Q.s to make 100%	81.38	81.18	80.98	81.68	84.55	82.78	80.38	79.78
Triethanolamine Q.s	Adjust pH up to 5.5–6.5							

**Table 2.** Composition of polymeric gel formulations.

Ingredients	ESI	ES2	ES3	ES4	ES5	ES6	ES7	ES8
Carbopol 940 (g)	1.5	1.5	1.2	0.85	0.75	1	1.5	2
Distilled water (mL)	100	100	100	100	100	100	100	100
Triethanolamine Q.s	Adjusted pH up to 5.5–6.5							

### Characterization of ebastine emulgel

#### Physical evaluation

The emulgels were inspected physically and visually for color change, phase separation, and consistency. The samples were preserved at different temperatures (8 °C, 25 °C, 40 °C, and 40 °C ± 75% relative humidity) for 28 days for any possible physical change (Burki et al., 2020).

#### Centrifugation study

The emulgel formulations were subjected to a centrifugation test. It was carried out by using a centrifuge at different temperatures (8 °C, 25 °C, and 40 °C). All the formulations were centrifuged by placing 2 g of a sample in a 15-mL centrifuge tube and rotating the tube at 3000 rpm for 30 min (Ali Khan et al., 2020).

#### Temperature swing test

A temperature swing test gives us an understanding of the stability of all emulgels at high and low temperatures (Ali Khan et al., 2020; Burki et al., 2020). All formulations were subjected to a freeze-and-thaw cycle for two days. One cycle was ‒4 °C for 8 h and 40 °C for 16 h. The stability was checked visually. Freeze-and-thaw cycle stability is used to measure the effect of temperature changes on an emulgel, as this process can accelerate changes. It also mimics potential outdoor storage conditions of final emulgel formulations.

#### pH determination

The pH of all of the fresh formulations was determined by a digital pH meter at different storage temperature conditions (i.e. at 8 °C, 25 °C, and 40 °C), and the pH values of all emulgels were recorded at regular intervals (i.e. 12, 24, 36, 48, and 72 h and 7, 14, 24, and 28 days).

#### Rheological studies

The viscosity of all freshly prepared formulations was checked using an NDJ-8S viscometer at a temperature of 25 °C, and the same process was repeated after every 12, 24, 36, 48, and 72 h and every 7, 14, 24, and 28 days.

#### Spreadability study

The spreadability coefficient was determined with an apparatus that had a wooden block and pulley attached at one end as per reported study of Ali Khan et al. (2020) with slight modification. On the basis of the slip and drag method, the spreadability of the prepared emulgel was calculated. The apparatus consisted of two glass slides: one of the slides was fixed on the wooden block and 2 g of the emulgel was applied on this fixed glass slide. The second glass slide was placed on the fixed slide. The emulgel was sandwiched between the fixed and upper slides of the same dimension. A 100-g weight was placed on the upper slide for 5 min to expel air and provide a uniform film of emulgel between the two slides. The upper glass slide was attached to the pulley through a hook and thread. At the other end of the thread attached to the upper slide some weight was applied, and this allowed the upper slide to slip on the fixed bottom slide. The time taken to separate the slides (in seconds) was recorded. The same procedure was repeated three times, and all the measurements for the formula given below calculated the spreadability of the emulgel.

(1) S = M × L/T

where *M* is the weight tied to upper slide, *L* is the length of glass slide, and *T* is the time taken to separate slides.

#### Drug content determination

One gram of the ebastine-loaded emulgel was dissolved in 10 mL of 80% ethanol; it was further diluted up to 100 mL with ethanol to obtain a clear solution (as a sample). This solution was subjected to UV spectrometry to determine the drug concentration. The absorbance was calculated at *λ* max 255 (Paul et al., 1987). The same procedure was repeated with the active drug (as a standard preparation). The drug concentration was calculated by the formula given below (Ali Khan et al., 2020):

(2) $$\text{\% of drug concentration }=\;\frac{\text{absorbance of sample}}{\text{absorbance of standard}}\;\times 100$$

#### *In vitro* drug release study

The *in vitro* drug release study was conducted according to the study of Khan et al. (2020), slightly modified. The Franz diffusion cell was used for *in vitro* drug release studies of the prepared formulation. The cellophane membrane was soaked in phosphate buffered solution overnight. The soaked cellophane membrane was placed between the donor and receiver compartments of the Franz diffusion cell. A specific amount of drug was applied on the cellophane membrane and stirred nonstop with a magnetic bar. After every hour, the sample was collected from the receptor chamber with the help of a sample syringe. The samples were analyzed spectrometrically at 255 nm, and the percentage of drug released was calculated.

#### DSC measurement

For the determination of the thermal stability of the ingredients in the emulgel, thermal analysis of the unloaded and prepared formulations was performed through DSC as per previous literature (Islam et al., 2021). The DSC of the individual ingredient and emulgel formulation was performed in a nitrogen environment applying gradually heat stress from ambient to 400 °C at the rate of 10 °C/min.

### *In vivo* anti-allergic activity

#### Animal selection

Fifteen adult male rabbits weighing 1–3 kg were selected and were divided into three groups, with each group containing five rabbits. These rabbits were kept under standard conditions as per the recommended environment. They were provided with adequate food, water, ventilation, temperature, and humidity.

#### Ethical approval

This study was approved by the Gomal University. Khan Ethical Committee, under reference number no. 851/QEC/GU. All the experiments were performed according to the guidelines of National Institute of Health (NIH).

#### Induction of allergy in animals

The allergy was induced in the experimental animals according to the study of Deng et al. (2016) with some modifications. Histamine hydrochloride solution (0.1 µg/mL in a solution of water/glycerol 50/50, v/v) was injected with a diabetic insulin syringe into the upper layer of the dorsal skin for the induction of allergy. The reactions were observed visually at different time intervals (erythema/redness). The scratching score and scores for no erythema, slight erythema, moderate erythema, and severe erythema were calculated after the injected allergen was observed on the rabbit skin.

#### Treatment protocols

The anti-allergic activity was assessed by applying the optimized formulation of emulgel on the dorsal skin of selected animals.

Rabbits were divided into three equal groups:

- Group A was treated with the ES3 emulgel formulation.
- Group B was treated with commercial product Benadryl^®^ (2% diphenhydramine hydrochloride).
- Group C was used as the control group and kept untreated.

### Statistical analysis

One-way ANOVA and Student’s *t*-test were applied to the data obtained using IBM SPSS version 20 (Armonk, NY) at level of significance of 5%. All the values, obtained from three independent experiments, were averaged, and presented as mean ± SD.

## Results and discussion

### Physical evaluation

The emulgels were assessed physically and visually for color (white viscous creamy), phase separation, and consistency. Eight different formulations (ES1–ES8) were evaluated for color, phase separation, homogeneity, and consistency. Almost all the formulations were stable. ES3 was found to be the most optimized formulation having white color, good appearance, excellent consistency, and no phase separation. The results are shown in Table 3.

**Table 3.** Physical evaluation of all formulations for color changes, phase separation, homogeneity, and consistency.

S/no.	Formulation code	Color	Phase separation	Homogeneity	Consistency
1	ES1	White	None	+++	+++
2	ES2	White	None	++	++
3	ES3	White	None	+++	+++
4	ES4	White	None	+++	+++
5	ES5	White	None	+	+
6	ES6	White	None	++	++
7	ES7	White	None	++	++
8	ES8	Off white	None	++	++

(+++) excellent; (++) good; (+) fair.

The optimized formulation, ES3, was studied at different conditions of temperature and humidity (i.e. 8 °C, 25 °C, 40 °C, and 40 °C ± 75% RH) in incubators for 28 days in order to observe any possible stability problem. The results were observed periodically (i.e. at 0 h, 24 h, 48 h, and 72 h and at 7 days, 21 days, and 28 days) as shown in Table 4.

**Table 4.** Evaluation of stability of formulation ES3 for duration of 4 weeks.

Stability parameters	Fresh	24 hours	48 hours	72 hours	07 days	21 days	28 days
Color	w	w	w	w	w	w	w
Odor	–ve	–ve	–ve	–ve	–ve	–ve	–ve
Phase separation	–ve	–ve	–ve	–ve	–ve	–ve	–ve

(w) white; (−ve) no change; (+ve) change.

### pH determination

The pH of a topical formulation is considered important for the stability of a preparation and its compatibility with skin (Ali Khan et al., 2020). It is an important factor, as it must range between 5 and 6 for suitable topical application of drugs and to avoid skin irritations (Islam et al., 2021). The pH of the optimized ebastine emulgel, ES3, was determined at different storage conditions (8 °C, 25 °C, and 40 °C), and the values were recorded at regular intervals. The pH of the ES3 emulgel was within the skin pH range (i.e. 5.5–6.5) as shown in Table 5. No significant changes (*p* > .05) in the pH values of the formulations were observed. These results show that the pH of the emulgels at different storage conditions was in the acceptable range and therefore the emulgels would probably not produce skin irritation. Hence, the ebastine emulgel preparation was suitable for topical applications.

**Table 5.** pH values for ES3 emulgel formulation at 8 °C, 25 °C, and 40 °C.

Temperature	Fresh	24 h	48 h	72 h	1 week	2 weeks	3 weeks	4 weeks
8 °C	5.2	5.2	5.3	5.3	5.3	5.4	5.5	5.5
25 °C	5.5	5.5	5.7	5.7	5.8	5.9	5.9	5.9
40 °C	5.6	5.6	5.6	5.7	5.9	6.0	6.0	6.03

### Temperature swing test and centrifugation study

**Figure 2.**
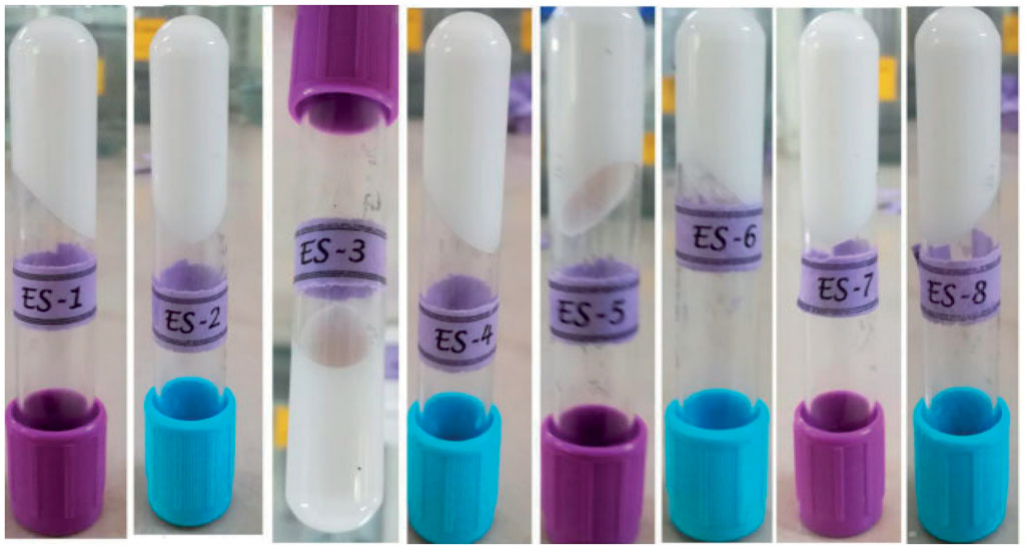
Physical appearance of emulgel formulation after temperature swing test.

The centrifugation test is vital for the determination of the stability of a topical formulation such as an emulgel and for the prediction of the shelf life of such a formulation. This test indicates the effect of physical stress on a semisolid preparation in terms of phase separation (Jadhav et al., 2010; Prakash et al., 2010; Deng et al., 2016; Islam et al., 2021). The prepared formulations of ebastine emulgels were passed through a physical test such as the freeze-and-thaw test and then centrifuged for stability. All formulations were found to be stable under different storage conditions (freeze-and-thaw cycle of ‒4 °C to 40 °C) and then they were centrifuged at 3000 rpm for 30 min. None of the formulations showed any signs of phase separation throughout the study period. Additionally, other stability parameters such as color and odor were also observed and were found to be stable (Figure 2).

### Rheological studies and viscosity

**Figure 3.**
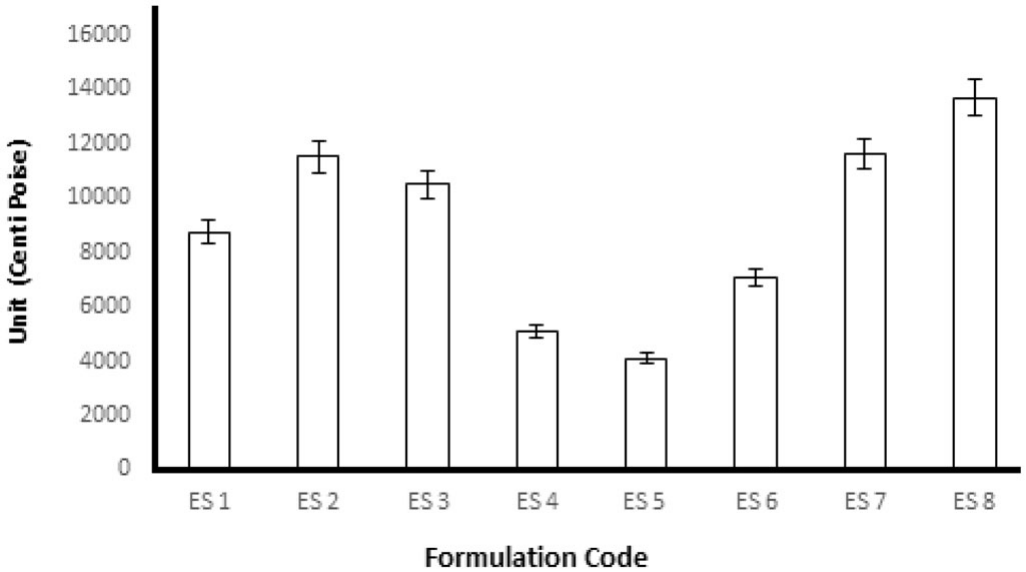
Viscosity of different formulations of emulgels in centipoise (cps).

Emulgel formulations with Carbopol had good viscoelastic properties, and their formulations had the possibility of returning to their original state once the stress is removed (Rohatagi et al., 2001). The rheological studies were performed for all formulations by using spindle 04 to measure the rheological behavior. About 0.5 g of the formulation to be tested was applied on the plate and left for equilibrium, and measurements were performed at 25 °C. The results obtained after the study of the fresh formulation and after 28 days are shown in Figure 3. The viscosity is an important parameter for a topical drug delivery system. It affects drug release and absorption at the site of action and thus affects the therapeutic benefit from the formulation (Khan et al., 2020). The rate of drug release decreases with an increase in the concentration of the gelling agent because the viscosity of the formulation increases (Shahi et al., 2013). In the present study, we observed that increases in the percentage of polymer led to a viscous emulgel (i.e. the viscosity of the emulgel depended on the concentration of polymer). The formulations from most viscous to least viscous were ES8 > ES2 > ES7 > ES3 > ES1 > ES6 > ES4 > ES5.

### Spreadability study

One of the most important criteria for a topical emulgel is reasonable spreadability. This feature can affect the therapeutic efficacy of topical preparations such as emulgels. Usually, the spreadability depends on the viscosity of the emulgel and the physicochemical properties of the polymers used in the preparation of emulgels. With an increase in the viscosity of a formulation, there is a decrease in its spreadability. Spreadability is defined as the extent of the area on which an emulgel readily spreads when it is applied to the skin (Kumar et al., 2014). A spreadability test was performed for all the formulations, and the spreadability was found to be in the range of 20.33–37.67. The spreadability values indicated that the emulgel was easily spreadable with the exertion of a small amount of shear. Overall, the spreadability of the emulgel formulations decreased with an increase in the concentration of the polymer. The spreadability is important because it indicates how the emulgel will behave when it is extruded from the tube. The spreadability of all emulgels from ‘more viscous and more time required for spreadability’ was ES8 > ES2 > ES7 > ES3 > ES1 > ES6 > ES4 > ES5, as shown in Table 6.

**Table 6.** Different characteristics of emulgel formulations.

Formulation codes	Viscosities (cps)	Spreadability	% Drug content	Drug release %
ES 1	8725 ± 7.78	27.09 ± 0.58	99.54 ± 1.54	69.84 ± 1.14
ES 2	11,510 ± 9.87	36.11 ± 0.58	98.97 ± 1.30	74.13 ± 1.24
ES 3	10,600 ± 9.90	29.67 ± 0.33	100.50 ± 1.98	74.25 ± 1.80
ES 4	5100 ± 4.50	22.01 ± 0.58	100 ± 1.56	62.84 ± 1.23
ES 5	4099 ± 3.45	20.33 ± 0.33	95.91 ± 1.67	65.12 ± 1.22
ES 6	7063 ± 6.78	23.67 ± 0.67	97.53 ± 1.98	64.13 ± 1.17
ES 7	11,625 ± 9.98	34.34 ± 0.58	101 ± 1.75	70.75 ± 1.77
ES 8	13,697 ± 10.23	37.67 ± 0.33	99.67 ± 1.60	74.67 ± 1.81

The data are presented as mean ± SD.

### Drug content

The uniform distribution of drugs in any pharmaceutical dosage form can be confirmed by percent of drug content (Khan et al., 2020; Islam et al., 2021). The drug content of all emulgel formulations was measured using a UV–vis spectrometer at *λ* max 255 nm. The content was found to be within the acceptable range of 95–105%. The results of the drug content revealed that % drug content fitted within the official limit (i.e. 100 ± 10%) allowed by the United States Pharmacopeia (USP). The results are reported in Table 6.

### *In vitro* drug release study

The therapeutic efficacy of any drug is dependent upon the release of drug from pharmaceutical preparation (Deng et al., 2016; Ali Khan et al., 2020; Khan et al., 2020; Islam et al., 2021). The release of drug from topical formulation depends upon several factors including gelling agents (several polymers), emulsifying agents (surfactants used), spreadability, and viscosity (Islam et al., 2021). The results showed that the release of ebastine from the emulgel was dependent on the concentration of Carbopol 940 in the formulation (i.e. the drug released decreased with an increasing concentration of the polymer) (Shahi et al., 2013). The drug release pattern of the formulations was ES8 > ES2 > ES7 > ES3 > ES5 > ES6 > ES4 > ES1. The drug released depended on the concentration of polymer; an increase in the concentration of polymer caused the drug release time to decrease and the diffusion through the membrane to decrease (Kumar et al., 2014). The results of our study were in agreement with Lauffer’s molecular diffusion theory of polymer gels, which states that the diffusion coefficient of a solute is inversely proportional to the volume fraction occupied by the gel-forming agent (Wiseman & Faulds, 1996).

**Figure 4.**
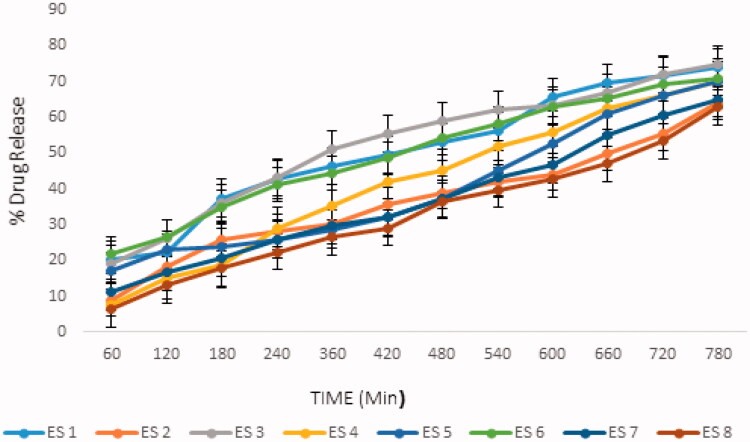
Percentage of ebastine released in emulgel formulations. The data were presented as the mean ± SD and the data were analyzed using ANOVA. *p*<.05 refers to statistical significance.

The percentage of drug released in all formulations was plotted against the time duration, as shown in Figure 4. The percentage of ebastine released from the ES1 to ES8 formulations after 12 hours of study is shown in Table 5.

### DSC measurements

**Figure 5.**
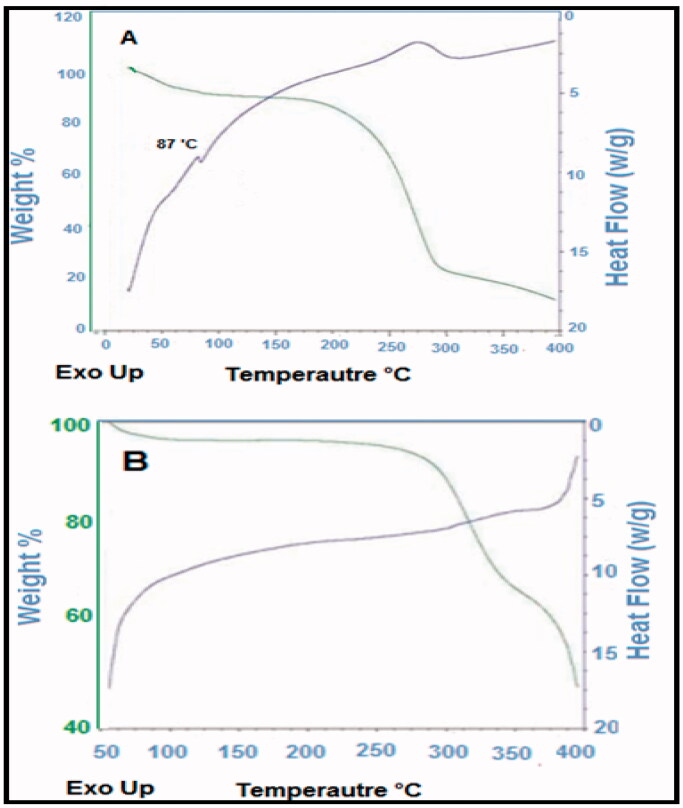
(a) DSC of ebastine and (b) emulgel formulation.

The thermal stability of the ebastine and prepared emulgel was analyzed through DSC, and results are presented in Figure 5(b). Through the thermogram, the glass transition temperature of the prepared formulations, i.e. emulgel was determined. The thermogram indicated a significant difference between the location of endothermic peak appeared near 90 °C for ebastine and that for drug-loaded formulation, i.e. ES3. The results indicated that formulations developed rigid polymeric network systems due to Carbopol which caused shifting of peaks toward an elevated temperature. This result is in agreement to the study reported in which the investigators fabricated sodium alginate‒PVA polymeric network systems and confirmed the grafting by indicating the shifting of exothermic peaks toward a higher temperature (Grattan et al., 2007).

### *In vivo* anti-allergic activity

In the present study, 15 adult rabbits were divided into groups A, B, and C, with each group having five rabbits. Group C was used as a control group without administration of any emulgel formulation. Group A was treated with the ES3 emulgel formulation. Group B was treated with Benadryl^®^. About 1 g of the emulgel was applied. The effects of the prepared emulgel were observed visually after a specified time period at different time intervals, such as 15, 30, and 60 min, and the results of groups A and B were compared with the results of group C (control group).

Two parameters, the erythema score and the scratching score, were noted at time intervals of 15, 30, and 60 minutes (Deng et al., 2016).

### Scratching score

**Figure 6.**
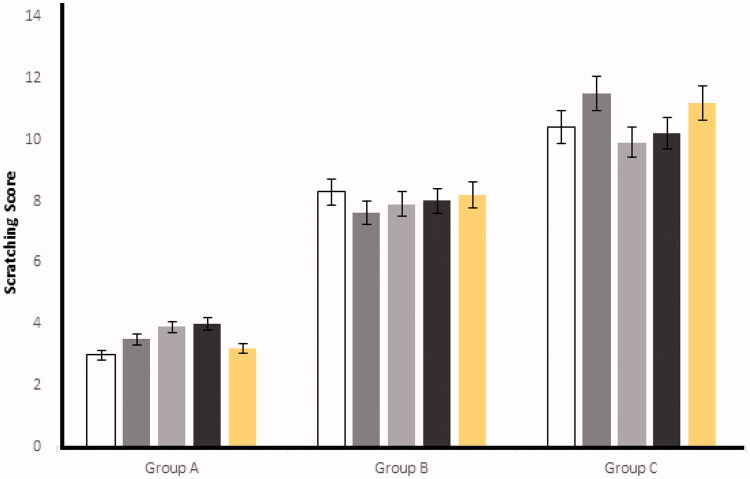
Effect of ES3 on the scratching score in rabbits. The data are presented as the mean ± SD and were analyzed using ANOVA. *p*<.05 refers to statistical significance of anti-allergic activity of ES3 and Benadryl^®^ from the control group.

**Figure 7.**
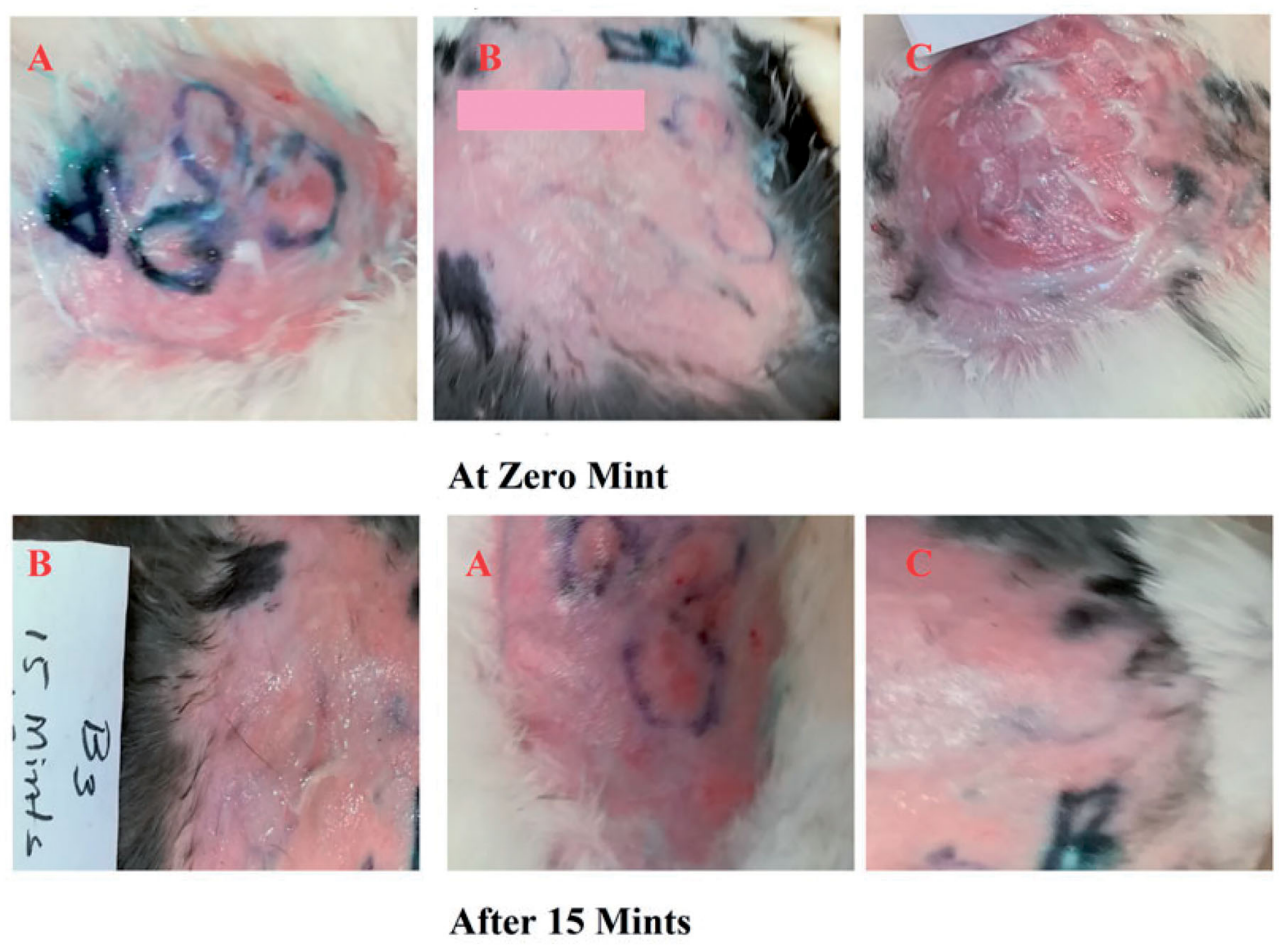
The effect of ebastine emulgel on group A and group B versus group C at 0 minute and 15 minutes.

After the injection of histamine at the experimental area, the rabbits started scratching because of a hypersensitive reaction (Deng et al., 2016). The scratching behavior was significantly (*p* < .05) reduced in the mean number of scratching/20 minutes by applying the optimized ES3 emulgel on group A and, similarly, on group B, when Benadryl^®^ was applied. On the other hand, control (group C) was kept untreated having significant higher (*p* < .05) numbers of scratching as compared to the groups A and B. The results for group A, group B, and group C are shown in Figures 6 and 7.

### Erythema score

After histamine injection, the development of erythema in all three groups was observed. The optimized formulation ES3 (1 g) was applied on the dorsal skin of group A rabbits and spread until it was completely adsorbed. The same procedure was repeated for group B by applying the Benadryl^®^. After 15, 30, and 60 minutes, the erythema score was significantly (*p* < .05) decreased from 4 to 0 (in group A) with the application of the ebastine-loaded emulgel (ES3) after 60 min, as compared to group B and group C.

**Figure 8.**
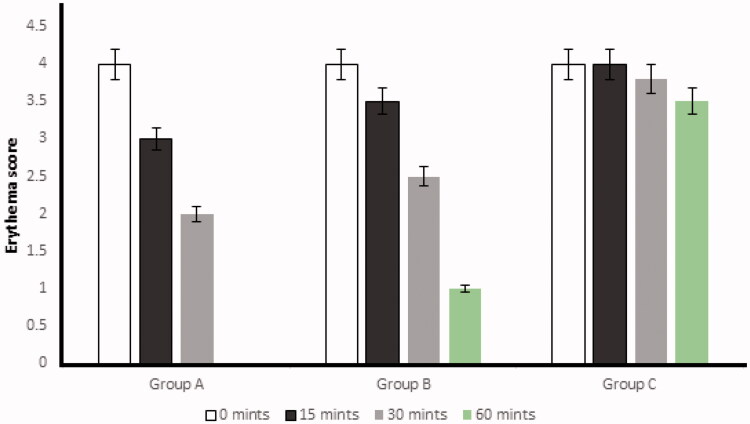
Effect of different formulations on the erythema score on the basis of a quantitative evaluation. The data are presented as the mean ± SD and were analyzed using ANOVA. *p<.05* refers to statistical significance of anti-allergic activity of ES3 and Benadryl^®^ from the control group.

**Figure 9.**
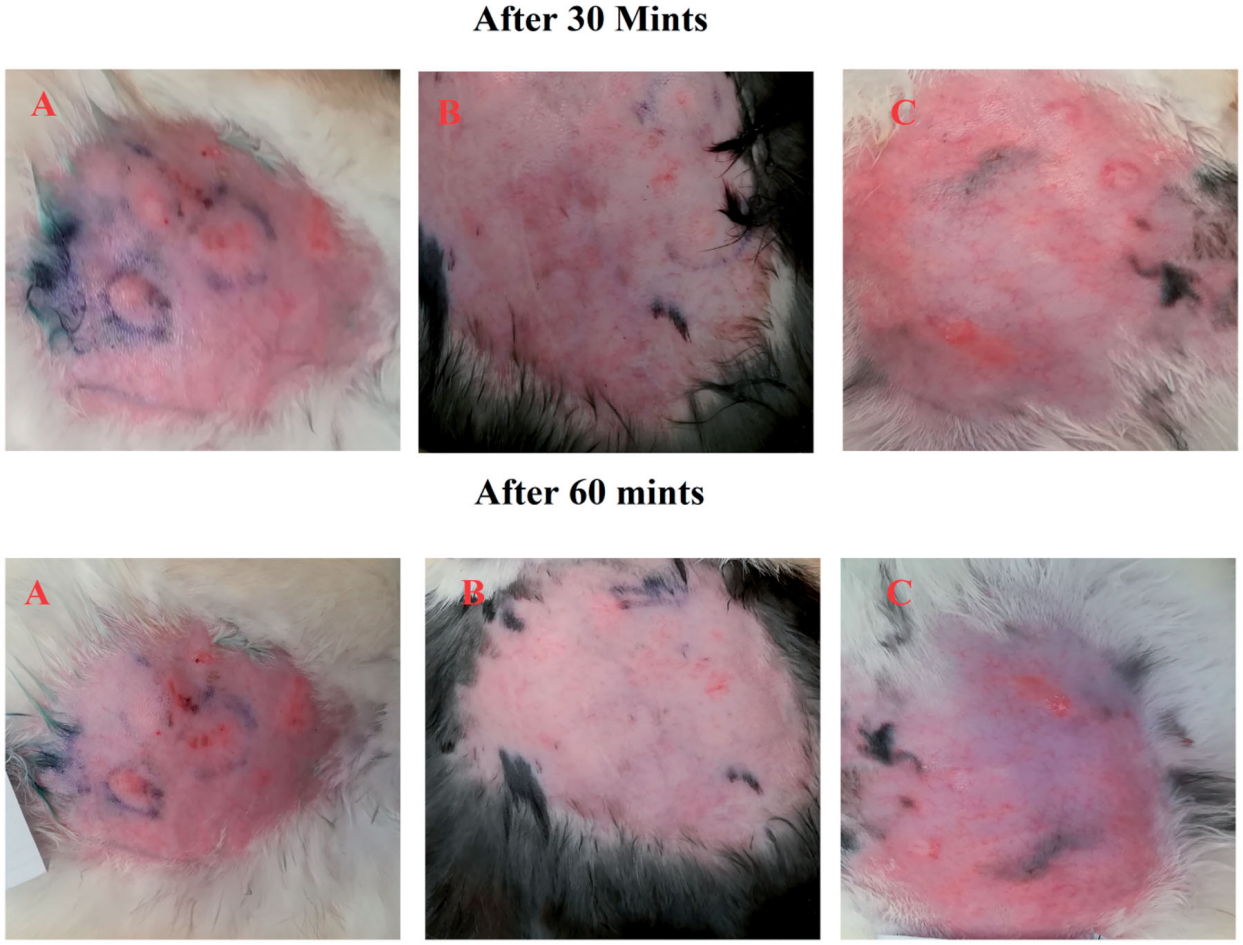
Effect of ebastine (emulgel) on group A and B versus group C after 30 min and 60 minutes.

The erythema score was calculated on the basis of the quantitative evaluation of the erythema that developed after histamine injection (0: no erythema; 1: slight erythema; 2: moderate erythema; 3: moderate to severe erythema; 4: severe erythema) in all groups. The application of the developed formulation on the dorsal skin of rabbits resulted in the absence of itching and erythema after 60 minutes compared with the control group (group C). This showed that the ebastine-loaded emulgel was effective for the treatment of skin allergies. The results have been presented in Figures 8 and 9.

## Conclusions

The topical drug delivery system is considered to be an important therapeutic technique for skin disorders. It minimizes the chances of systemic toxicity and provides a quick onset of action compared with oral therapy. Ebastine is a second-generation H1 antagonist antihistamine drug that is prescribed for all types of allergies. It is especially the drug of choice for urticaria and allergic rhinitis. To overcome the side effects associated with oral ebastine therapy and to achieve benefits related to topical therapy, ebastine topical gels were prepared and evaluated successfully. From the results of the study, we concluded that an ebastine emulgel formulation prepared with Carbopol 940 showed acceptable physical characteristics upon storage for 28 days. The prepared emulgel showed excellent anti-allergic activity and high drug release when compared with the marketed cream. Therefore, ebastine emulgel can be used as topical anti-allergic therapy for the treatment of urticaria.

## Data Availability

Not applicable.
